# Pathological Conditions Produced by Galvanic Action between Dissimilar Metals in the Treatment of Caries of the Teeth

**Published:** 1891-10

**Authors:** George W. Whitefield

**Affiliations:** Evanston, Ills.


### PATHOLOGICAL CONDITIONS PRODUCED BY GALVANIC ACTION BETWEEN DISSIMILAR METALS IN THE TREATMENT OF CARIES OF THE TEETH.[1]

BY GEORGE W. WHITEFIELD, M.D., D.D.S., EVANSTON, ILL.

To arrest the causes that destroy the teeth, fillings of different kinds have been and are being used to repair the loss of continuity of tooth-structure, to protect exposed parts, and restore the contour of the organ. Prominent among these filling-materials are gold, tin, cements, and amalgam. Amalgam is composed of varying proportions of tin and silver, with a small amount of gold or platinum amalgamated with quicksilver. There are also amalgams of copper, and some containing zinc.

In a general way, let me now introduce the subject of the all-pervading mysterious form of vibration we call electricity, for this will be necessary to comprehend the galvanic action produced in the mouth by dissimilar metals. Different qualifying names are employed to designate the kind of electrical manifestations, as mag-

[1] Read before Oral Surgical Section of American Medical Association, Washington, D. C., 1891.

netic, frictional or static, galvanic, voltaic or dynamic, and thermal electricity.

We find that all molecular disturbances give rise to disturbed electrical equilibrium; all motion tends to produce this condition. We notice the result only when the conditions are favorable; that is, when the conditions are such that the equilibrium is not too easily re-established.

Electricity is termed positive (+) when it has an accumulation of electrical force to impart, negative (—) when it will require such an amount of electrical energy to restore the electrical equilibrium. The difference of electrical conditions is termed the difference of potential.

Difference of potential is a difference of electrical conditions, by virtue of which work is done by the electrical vibrations in moving from the point of higher to that of a lower potential, and is measured by the unit of quantity of electricity thus transmitted.

The passage of electricity from the point of higher to that of lower potential is termed electrical current, and the bodies along which it passes are conductors. The opposition to the passage of the current is termed the resistance.

In order to understand the action of what is called the electric current, I will give the familiar illustration of two reservoirs of water connected by a pipe. The electric current may be likened to the flow of water through this pipe from the higher to the lower level. The unit of current strength, also called the rate of flow or intensity, is the ampère. In the illustration we would say the water is flowing through the pipe at the rate of one gallon per second. In speaking of the electric current, we would say it has a strength of, say, one ampère.

The unit of electromotive force, also called electrical pressure, or tension, or difference of potential, is the volt. In the illustration the head of water, or the difference between the levels A and B, is similar to the electromotive force.

The unit of resistance is the ohm. Resistance may be compared with the friction of the internal surface of the pipe offered to the water. It follows that the more cross-section the less friction, and the less cross-section the more friction, for the same volume of water flowing through the pipe in a given time.

Conductors are good or bad as they convey or resist the passage of the electrical current; different metals vary in their conductivity, silver and copper being high in the scale, while German silver and steel retard the current; moisture is a good conductor, while glass, kerite, rubber, and dry air are among the poorest conductors: the resistance varies with the length of the circuit, the conductivity and cross-section of the conductors.

As the current is the same strength throughout its entire length, it naturally follows that the larger the conductors, other things being equal, the less the resistance, and, on the other hand, the less the surface or poorer the conductor, the less the current and reduced activity of the battery. When the current is too great for the conductors the wave length is shortened to that of heat, which varies according to the current and the conductors. The wave length may be shortened to that of light vibrations so that the conductors may glow as in the incandescent lamp or cautery snare. Heat generated in this way is now employed in electric welding and for the fusing of metals requiring a high temperature, such as platinum, iridium, etc.

Magnetism derives its name from the place where magnetic ore was first found,—Magnesia, Asia Minor.

Frictional or static electricity is of but little interest to us as a profession, although often, in a dry atmosphere, it may be a very annoying companion by the dental chair, when each movement of the operator produces by friction a difference of potential between the operator and the patient; then if the discharge is by way of an instrument to a filling, or moisture in a cavity of a tooth, the result, to say the least, is unpleasant, and often quite startling if the patient, by jumping, upsets the instruments.

Frictional electricity is to galvanic electricity what a red-hot needle would be to a large, moderately-heated crow-bar: the crow-bar contains more heat than the needle, but one has volume, the other intensity. Frictional electricity is so intense that it will jump through air, the length of the spark being governed by the intensity of the current.

Galvanic electricity, on the contrary, will traverse the circumference of the globe rather than leap a short space. Galvanic, voltaic, or dynamic electricity is the particular manifestation of this force, most interesting in a practical way to the dentist. The current derived from the battery is called galvanic or voltaic, in honor of the experimenters who, less than one hundred years ago, discovered the method of generating and collecting the force through chemical action. It is this manifestation of electrical energy that is employed in the arts, consequently the name dynamic.

Where the extensive use of electricity is required, the dynamo is employed for economical reasons. Batteries consist of positive (+) and negative (—) elements, immersed in an exciting fluid, or fluids. The fluid employed varies according to the construction of the battery and kind of work to be done, being acid or alkaline; though even pure water will generate a current, if two metals are immersed in it, where one metal is more readily oxidized than the other. (Note the fact that action would take place if the elements were composed of two samples of amalgam, if one was composed of less oxidizable metals than the other.)

The positive (+) element of the battery is the one on which the exciting fluid acts; that is, the fluid destroys the positive (+) element, molecule by molecule, while the negative (—) element should sustain no loss of structure. If the negative (—) element is acted upon by the fluid, counter-currents are generated which interfere with the usefulness of the battery. I spoke of the resistance of the conductors: the fluids of the batteries impose resistance, termed the internal resistance. The electrical energy of a battery is the energy of the current, less the internal resistance, and the resistance of the circuit. The resistance, both internal and of the circuit, modify the action of the battery. If the elements are connected with wire of sufficient size to convey the current, the electromotive force will be reduced in proportion, as the length of the wire is increased, or the size (cross-section) of the wire is reduced; while, if the elements touch in the fluid, the action is most violent, and the batteries soon become polarized; that is, the surface of the elements becomes so charged that the fluid ceases to affect them, or the fluid becomes so saturated with the waste product of the combustion of the positive (+) element that action practically ceases, unless provision is made for it by having the fluid constantly renewed, as in some kinds of batteries designed for constant work.

When the circuit is opened shock is felt, if the current is of sufficient strength: this shock is the result of the accumulative energy being suddenly discharged; different devices are made use of to produce this result, such as the induction coil, constructed with a vibrator, which rapidly makes and breaks the circuit; an example of this is the medical battery, and the Ruhmkorff coil: the current produced in this way is called the Faradic.

Now, to apply the foregoing to the battery formed by dissimilar metals in the mouth, where the fluids of the mouth are the exciting media, gold will be the negative (—) element, as the fluids of the mouth have no action on this metal; the positive (+) element is the base metals, whether used separately, as tin, or, as most commonly, the combination of mercury with tin, silver, zinc, and copper, as in the amalgams in use.

With gold and tin to form the voltaic pair, the base metal soon becomes coated, and the current practically ceases; but with amalgam, the mercury performs the same office in the mouth that it does in the laboratory,—it presents the metal in a form that is easily acted upon by the exciting fluid.

We always have good conductors in the fluids of the mouth, containing, as they do, mucus and various earthy salts, while often the fillings touch in the same tooth, or as approximal fillings of gold in one tooth, and amalgam in another.

Resistance varies in each individual case, according to the character of the secretions, or the situation of the fillings.

The action will vary according as the conditions are changed: naturally, where food is left between the teeth to decompose, the acid resulting from such fermentation will form a more exciting media than normal alkaline saliva. Where a filling is left rough and jagged, overhanging and irritating the gums, it presents an exaggerated surface to be acted upon; besides, by irritating the gums, it causes a secretion from them that forms an excellent exciting fluid. This is, unfortunately, the too frequent result of careless operating.

Now, what of all this? Is it only theory? Are these statements founded on scientific facts? Can they be established by proof?

The *Western Electrician* of February 11, 1888, says, in reference to a talk given before the Chicago Electrical Club:

"Dr. Whitefield succeeded in settling a question that has been in dispute among dentists as to the electric action of amalgam and gold fillings in the same tooth, or even in the same mouth.

"That a current was generated by amalgam and gold fillings when placed in water, even when they were insulated from each other, was conclusively shown by bringing a galvanometer into circuit. The point is one that may be combated, but the evidence produced by the galvanometer cannot be controverted."

Now, if there is galvanic action, what harm can it do? Let us quote from an article on amalgam in the November *Dental Review*, 1887:

"Now the question arises,—Shall we ever use amalgam and gold in the same tooth, and if so, why? and if not, why not? There has been such varied teaching in the past in regard to this subject that it is time the old errors should be exposed and scientific teaching presented to our students of to-day. The old way was to put both amalgam and gold in the same tooth if it seemed necessary, but never to let them touch each other, or grave results would follow. After seeing such methods pursued for a term of years we find that, in spite of the teachings, grave results did follow, which ultimately resulted in the loss of the gold filling, while the amalgam remained comparatively sound, or, rather, the tooth structure surrounding the amalgam filling."

This is correct, but let us review the writer's following statements:

"We have found that if the two metals touch in the electric battery there is no longer a current,—it is dead, no shock is produced, and it is just the same when the metals are in a tooth in the presence of an acid as it is out of the mouth in the laboratory. Therefore, we found, first from an unpleasant experience and later from theory, that if amalgam and gold are placed in the same tooth they should have an uninterrupted communication, should be in complete apposition, so that no electric or galvanic action can take place because they touch each other."

Let us consider these statements and see if they harmonize with the laws of physics. Now, if the elements touch (the fillings) there is practically no resistance in the circuit; consequently, the battery's action is most violent, up to the capacity of the battery to generate current.

The writer says no shock is produced. He is right; the current flows evenly through the short circuit, so the equipoise is practically maintained, and shock is produced only when the current is interrupted, when an accumulation of energy is suddenly discharged.

Let me try to point out what has misled my friend into making these erroneous statements:

If gold and amalgam touch in the same tooth there is practically less destruction around the gold filling. This is easily explained, as the galvanic action is so violent that the surface of the amalgam filling is soon destroyed; that is, all the baser metals are consumed from the face of the plug, leaving the silver, gold, or platinum of which it is composed on the surface, which practically changes the amalgam to nearly a silver surface, thus raising it to nearly a negative metal, while, besides this, the coating protects even the surface of the silver.

This statement holds true with regard to very old plugs of amalgam: their surface is no longer amalgam, it is negative metal.

But at what fearful cost are such old fillings raised to the dignity of negative elements!

Let us now note some of the harmful phases of this subject.

First, galvanic action has a tendency to accelerate the blood flow, producing hyperæmia and hyperæsthesia, and in some cases violent nervous phenomena. This is especially the case where, from the situation of the fillings, the energy is accumulated, then suddenly discharged, producing shock, as each can demonstrate by touching a bit of zinc or the blade of a penknife to a filling in your own mouth.

Secondly, the current is generated from all parts of the elements that come in contact with moisture and are not protected by a coating. The portion of the amalgam filling that are protected by tooth-structure becomes so coated that there is practically no action except on its exposed surface; on the contrary, gold remains bright on all its surfaces, and, as moisture pervades the whole tooth, no matter how well the filling is inserted, moisture will reach it by way of the intertubular spaces; consequently electrolytic action can take place from all portions of the gold element, naturally causing considerable destruction around the gold filling. And this is not all. Among the commonest elements found in the mouth is chloride of sodium (salt); galvanic action readily breaks up this compound: the chlorine liberates oxygen and unites with the hydrogen of the water, forming hydrochloric acid, leaving the oxygen in its most active state. Other acids may be formed in this way, such as sulphuric, nitric, etc. The electrolytic action and the acids thus formed are sufficient to roughen the surface of the teeth to give lodgement to colonies of "microbes."

Chlorine in its nascent state will readily unite with the mercury of the amalgam, and the chlorides of mercury may be formed in sufficient quantities to produce symptoms of mercurial poisoning in those susceptible to its influences; these salts of mercury may also explain the immunity from decay of teeth stopped with ill-fitting amalgam plugs, the germicidal effects of the mercury being sufficient to prevent colonization by micro-organisms, and consequent destruction of the tooth.

The oxygen might, from peculiarities of the individual case, unite with other salts than those of the teeth, and the same with the acids. We all know that the neglect some mouths get would be total destruction in others. The elements that usually produce decay seem to be inert in their case, and the same condition undoubtedly explains why electrolytic action that would destroy in one case is apparently harmless in others, although such cases are in the minority.

The usual result of placing amalgam in the back teeth, while gold is placed in the front teeth, of children, will explain why the gold fillings have to be renewed so often ; also, that in the electrical action is a clue to the oft-repeated tale brought us by the laity that amalgam stands better than gold, as the amalgam still remains, and the gold fillings, that have been replaced several times, are loose again.

If amalgam must be used, use those grades that will readily become coated, as the coating will reduce the galvanic action, protecting the plug from the fluids of the mouth, except where attrition and brushing keep them bright. Gold and amalgam—in fact any metal—should be avoided for teeth that are of such soft structure that the pressure produced in inserting gold would tend to break down the tubuli, while metals never stand well in teeth of very soft structure ; in fact, you can as soon expect to prevent water flowing under the edge of a board resting on the ends of the blocks in cedar-block pavement in the street as to expect to exclude moisture from a soft tooth with a metallic filling, as moisture is bound to penetrate between the tubuli. Even the normal plasma exuding from the tubuli will have a tendency to assist the destruction of the hard structure when it becomes vitiated, as it must in the temperature of the mouth, as it is as foreign a substance when lost from the tubuli as is the blood that weeps from abraded tissue.

The cements and gutta-percha are the only filling-materials that should be used in such teeth. If you will use these materials, in the course of eighteen months or two years the tooth will be found quite hard and capable of proper manipulation to prepare it for the king of metals and the king of filling-materials,—gold. A tooth treated in this way will not be as sensitive, and the fillings will last.

